# Vitamin K1 enhances sorafenib-induced growth inhibition and apoptosis of human malignant glioma cells by blocking the Raf/MEK/ERK pathway

**DOI:** 10.1186/1477-7819-10-60

**Published:** 2012-04-21

**Authors:** Wei Du, Jing-ru Zhou, Dong-liang Wang, Kai Gong, Qing-jun Zhang

**Affiliations:** 1 Department of Neurosurgery, Peking University People’s Hospital, No. 11 Xizhimen South Street, Beijing, 100044, China

**Keywords:** Apoptosis, Glioma, MAPK, Sorafenib, Vitamin K1

## Abstract

**Background:**

The combined effects of anticancer drugs with nutritional factors against tumor cells have been reported previously. This study characterized the efficacy and possible mechanisms of the combination of sorafenib and vitamin K1 (VK1) on glioma cell lines.

**Methods:**

We examined the effects of sorafenib, VK1 or their combination on the proliferation and apoptosis of human malignant glioma cell lines (BT325 and U251) by 3-(4,5-dimethylthiazol-2-yl)-2,5-diphenyl tetrazolium bromide (MTT) assay, flow cytometry and 4′,6-diamidino-2-phenylindole (DAPI) assay. The signaling pathway changes were detected by western blotting.

**Results:**

Sorafenib, as a single agent, showed antitumor activity in a dose-dependent manner in glioma cells, but the effects were more pronounced when used in combination with VK1 treatment. Sorafenib in combination with VK1 treatment produced marked potentiation of growth inhibition and apoptosis, and reduced expression of phospho-mitogen-activated protein kinase kinase (MEK) and phospho-extracellular signal-regulated kinase (ERK). Furthermore, the expression levels of antiapoptotic proteins Bcl-2 and Mcl-1 were significantly reduced.

**Conclusions:**

Our findings indicated that VK1 enhanced the cytotoxicity effect of sorafenib through inhibiting the Raf/MEK/ERK signaling pathway in glioma cells, and suggested that sorafenib in combination with VK1 maybe a new therapeutic option for patients with gliomas.

## Background

Malignant gliomas are the most prevalent primary brain tumors in adults, exhibiting a high rate of cell proliferation and migration activities [1]. Despite tremendous efforts in the improvement of therapeutics, such as surgery, radiotherapy and chemotherapy, the clinical outcome of gliomas remains dismaying [2].

Recent publications indicate that the majority of gliomas display upregulated Raf kinase [3], which is an essential serine/threonine kinase constituent of the mitogen-activated protein kinase (MAPK) pathway. Raf is recruited to the cellular membrane and activated by GTP-bound activated Ras. Activated Raf phosphorylates MAPK kinase 1/2 (MEK 1/2) [4], which in turn phosphorylates and activates extracellular signal-regulated kinase 1/2 (ERK 1/2). Then, ERK activation leads to phosphorylation of a variety of transcription factors and results in induction of gene expression and proliferation [5]. The upregulation of the Raf/MEK/ERK pathway has been proven to take part in the amplification of mitogenic stimuli and promotion of cellular proliferation of malignant gliomas. Therefore downregulation of the Raf/MEK/ERK pathway may be a valuable therapy for glioma patients [6].

Sorafenib (Nexavar, BAY 43–9006) is an oral, small molecule multikinase inhibitor that was originally developed as a Raf kinase inhibitor [7]. Subsequent studies have demonstrated that it also inhibits receptor tyrosine kinases, including vascular endothelial growth factor (VEGFR) and platelet-derived growth factor receptor (PDGFR) [8]. Sorafenib has shown a preclinical antitumor effect against a variety of tumor types and has been approved by the US Food and Drug Administration (FDA) for the treatment of advanced renal carcinoma and unresectable hepatocellular carcinoma [9-11]. Whereas sorafenib treatment of glioma cell lines and tumor xenografts results in cell growth inhibition and tumor growth regression [12,13], its use in the clinical treatment of patients with malignant gliomas has yielded disappointing results [14]. Despite its relative ineffectiveness in patients with malignant gliomas, the ability of sorafenib to inhibit tumor cell proliferation suggests that it may be useful in combination with other therapeutic agents.

Vitamin Ks are fat-soluble vitamins that are involved in bone metabolism and blood coagulation [15]. There are two natural forms of vitamin K, vitamin K1 (VK1, mostly found in green leafy vegetables) and vitamin K2 (VK2, synthesized by the intestinal flora). It has been shown that vitamin Ks could inhibit the growth of various types of cancer cells *in vitro* as a single agent or in combination with other chemotherapy [16,17]. Recent studies have shown that VK1 can enhance the effects of sorafenib-mediated hepatocellular carcinoma cell growth inhibition through inhibiting the density-enhanced phosphatase 1 (DEP-1)-regulated c-Met-Akt pathway [18]. However, the combinational effect of sorafenib and VK1 on glioma cells has not been studied so far. In this work, we employed the human malignant glioma cell lines BT325 and U251 to evaluate the induction apoptosis and inhibition of cell proliferation of sorafenib in combination with VK1 through the Raf/MEK/ERK signaling pathway.

## Methods

### Cells and reagents

The BT325 cell line was obtained from Beijing Neurosurgical Institute Collection and the U251 cell line was bought from American Type Culture Collection (Manassas, VA, USA). All cells were cultured in Dulbecco’s modified Eagle medium (DMEM) containing 10% fetal bovine serum (FBS) at 37°C and 5% CO_2_. Sorafenib was purchased from Bayer Corporation (West Haven, CT, USA) and dissolved in dimethylsulfoxide (DMSO) with cell medium to the given concentration with a final DMSO concentration of 0.1%. VK1 was purchased from Sigma-Aldrich Chemical, and dissolved in 99.5% ethanol at a stock concentration of 100 mmol/l and then diluted to appropriate concentrations with medium. DMSO or ethanol was added to medium at 0.1% (V/V) as a solvent control.

### Cytotoxicity assay

BT325 and U251 cells were plated at a density of 5 × 10^4^ cells/ml in 96-well plates (Corning, USA) for 24 h. Then the medium was replaced with fresh DMEM containing various concentrations of sorafenib, VK1 or combination of the two agents for 72 h. Cells were washed twice with phosphate-buffered saline (PBS), and 20 μl 3-(4,5-dimethylthiazol-2-yl)-2,5-diphenyl tetrazolium bromide (MTT) solution (5 mg/ml) was added to each well. After 4 h incubation at 37°C, the culture containing MTT was carefully removed, 200 μl of DMSO was added to each well, and absorbance at 570 nm was measured using MRX II absorbance reader (DYNEX Technologies, Chantilly, VA, USA). The cell viability was assessed by the percentage of absorbance in cells at least three independent tests.

### Apoptosis analysis by flow cytometer

Annexin V-fluorescein isothiocyanate (FITC)/propidium iodide (PI) kit (BD Biosciences, Sparks, MD, USA) was used to measure the percentage of apoptosis induced by sorafenib and VK1. Cells were cultured in six-well plates at 3 × 10^5^ cells per well and treated with the agents for 10 h. The cells were harvested, washed with cold PBS, and then resuspended in 500 μl of binding buffer. A total of 5 μl of annexinV-FITC solution and 10 μl PI (1 μg/ml) were added to these cells for 30 minutes away from the light. Using flow cytometer (Becton Dickinson, USA) to detect apoptosis through channels two and three. In all, 10,000 cells were collected for each sample.

### 4′,6-Diamidino-2-phenylindole (DAPI) assay

Cells were cultured on chamber slides and treated with sorafenib, VK1 or their combination. Then, 24 h later, cells were washed with cold PBS and stained with DAPI for 10 minutes away from the light. Nuclear morphological changes were examined using fluorescence microscopy (DFC480; Leica Microsystems, Germany).

### Western blotting

Cells were plated in tissue culture dishes overnight and treated with the agents for 24 h. After harvest, the cells were resuspended in lysis buffer (150 mM NaCl, 50 mM Tris–HCl, pH 7.4, 2 mM ethylenediaminetetra-acetic acid (EDTA), 1% NP-40) containing protease inhibitor cocktail (Amresco, Solon, OH, USA). Equal amount of total protein extracts were separated by 10% standard sodium dodecyl sulfate polyacrylamide gel electrophoresis (SDS-PAGE) and transferred onto a polyvinylidene fluoride (PVDF) membrane (0.45 mm, Millipore, Bedford, MA, USA). Non-specific antibody binding was blocked with 5% fat-free dry milk/Tris-buffered saline (TBS)-Tween 20 (TBST) at room temperature for 1 h. The membrane was then incubated overnight at 4°C with the following specific antibodies to MEK, ERK, phospho-MEK, phospho-ERK, Bcl-2, Mcl-1, Bcl-xl, Bax and β-actin (Cell Signaling Technology). Horseradish peroxidase-linked anti-mouse or anti-rabbit IgG were then used as secondary antibody, and bands were detected with enhanced chemiluminescence reagent (Amersham Bioscience, Piscataway, NJ, USA).

### Statistical analysis

Data were expressed as means ± standard deviation (SD). Statistical analyses were performed using one-way analysis of variance (ANOVA) via SPSS 13.0 software (SPSS,Chicago, IL, USA). A value of *P*<0.05 was considered statistically significant.

## Results

### Inhibition of glioma cell growth by sorafenib plus VK1

**Figure 1:**
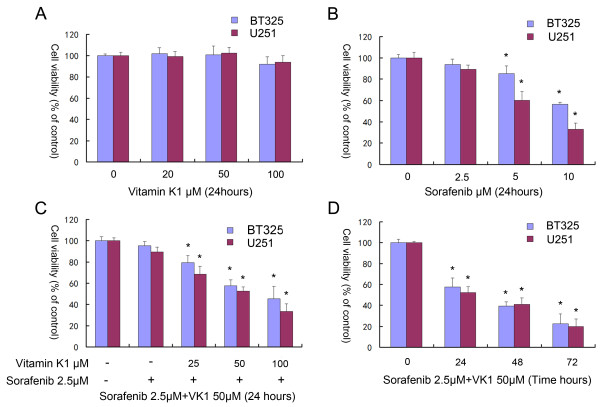
**Effect of vitamin K1 (VK1) or sorafenib, or their combination, on cell viability of glioma cells. (A)** BT325 and U251 cells were incubated with 0, 25, 50 or 100 μM VK1 for 24 h. **(B)** BT325 and U251 cells were incubated with 0, 2.5, 5 or 10 μM sorafenib for 24 h. **(C)** Cells were incubated with sorafenib (2.5 μM) plus varying concentrations of VK1 (0, 25, 50, 100 μM) for 24 h. **(D)** Cells were incubated with sorafenib (2.5 μM) plus VK1 (50 μM) for 24, 48, 72 h. The cell viability was evaluated by 3-(4,5-dimethylthiazol-2-yl)-2,5-diphenyl tetrazolium bromide (MTT) assay. Cell viability was expressed as the percentage of cell survival compared with the control. Data were repeated at least three times. An asterisk indicates *P*<0.05 vsthe control vehicle (A,B,D), or sorafenib-induced cells without VK1 treatment (C).

In order to investigate the impact of sorafenib plus VK1 on the proliferation of glioma cells, we exposed BT325 and U251 cells to the studied agents, either individually or in combination. MTT assays indicated that VK1 is safe for use in cells up to 100 μM compared to the vehicle control for 24 h (Figure 1A), and sorafenib (0, 2.5, 5, 10 μM) exerted a dose-dependent cell growth inhibition (Figure 1B). To study the interaction of sorafenib and VK1, we evaluated sorafenib (2.5 μM) in combination with VK1 (0, 25, 50, 100 μM) for 24 h in BT325 and U251 cells, which resulted in a dose-dependent manner inhibition of cell growth (Figure 1C). The time-dependent cell growth inhibition of sorafenib (2.5 μM) plus VK1 (50 μM) on BT325 and U251 cells is shown in Figure 1D.

### Induction of glioma cell apoptosis by sorafenib plus VK1

**Figure 2:**
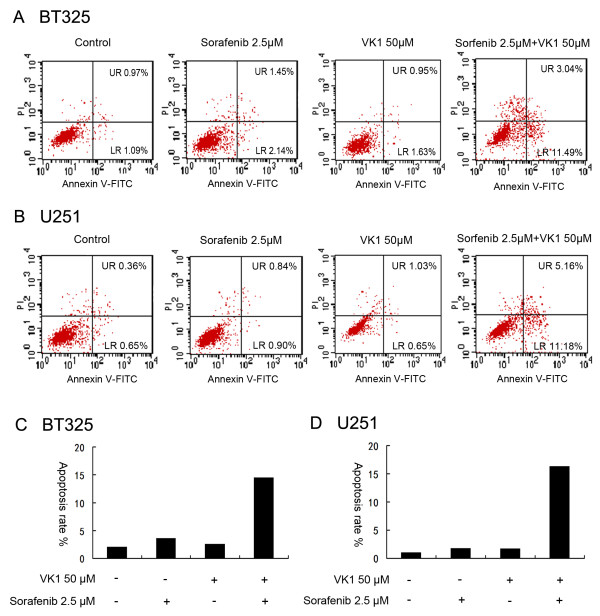
**Sorafenib in combination with vitamin K1 (VK1) induces apoptosis in BT325 (A,C) and U251 (B,D) cells.** Cells were treated with sorafenib (2.5 μM), VK1 (50 μM) alone or in combination for 10 h. Then, cells were harvested and stained with annexin V-fluorescein isothiocyanate (FITC)/propidium iodide (PI) followed by flow cytometry analysis. The four different cell populations marked as: unstained cells indicating a live cell population (LL, lower left), annexin V-positive and PI-negative stained cells indicating early apoptosis (LR, lower right), annexin V/PI double-stained cells showing late apoptosis (UR, upper right), and annexin V-negative and PI-positive stained cells showing dead cells (UL, upper left).

Apoptosis is the major consequence of tumor cells exposed to chemotherapy agents. We investigated whether this combination induced apoptosis in BT325 and U251 cells. Cells were incubated with sorafenib (2.5 μM) and VK1 (50 μM) individually or in combination for 10 h, and examined by annexin V-FITC/PI staining. As shown in Figure 2, the four quadrants in each panel correspond to: necrotic cells (upper left), apoptotic late cells (upper right), apoptotic early cells (lower right), viable cells (lower left). The results indicated that neither 2.5 μM sorafenib nor 50 μM VK1 induced significant apoptosis as a single agent, but the combination treatment for BT325 and U251 cells resulted in 14.5% and 16.3% apoptosis of cells, respectively.

**Figure 3:**
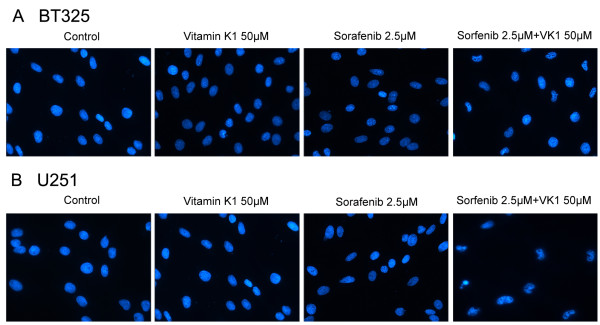
**4′,6-Diamidino-2-phenylindole staining of BT325 (A) and U251 (B) cells treated with sorafenib (2.5 μM) plus vitamin K1 (VK1) (50 μM).** Cells were treated with sorafenib, VK1 or their combination for 24 h. The cells treated with the combination of the agents showed morphological changes of apoptosis, including a condensed and fragmented nuclear structure and decreased cell size (original magnification 400×).

In addition, BT325 and U251 cells were incubated with sorafenib (2.5 μM) and VK1 (50 μM) individually or in combination for 24 h, and then nuclei of the cells were stained with DAPI. As shown in Figure 3, compared with either agent alone, the staining of combination treated cells showed the presence of characteristic apoptosis, including a condensed and fragmented nuclear structure and decreased cell size.

### Inhibition of the Raf/MEK/ERK pathway by sorafenib plus VK1

**Figure 4:**
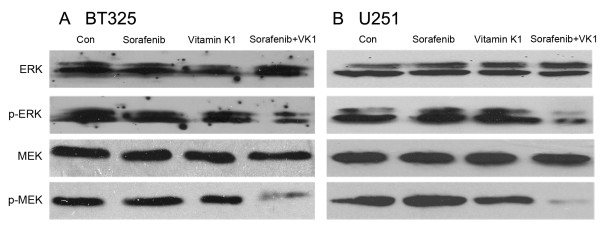
**Sorafenib in combination with vitamin K1 (VK1) inhibits phospho-mitogen-activated protein kinase kinase (MEK) and phospho-extracellular signal-regulated kinase (ERK) in BT325 (A) and U251 (B) cells.** Cells were treated with sorafenib (2.5 μM), VK1 (50 μM), or their combination for 24 h.

As the Raf/MEK/ERK signaling pathway plays an important role in glioma cell growth, we assessed the expression of phospho-MEK and phospho-ERK by western blotting. BT325 and U251 cells were treated with the agents for 24 h. For these studies, neither 2.5 μM sorafenib nor 50 μM VK1 alone inhibited phospho-MEK and phospho-ERK, but the combination of agents dramatically reduced the phosphorylation levels of both MEK and ERK (Figure 4). Total MEK and ERK levels were not changed.

### Involvement of sorafenib plus VK1-mediated apoptosis of glioma cells

**Figure 5:**
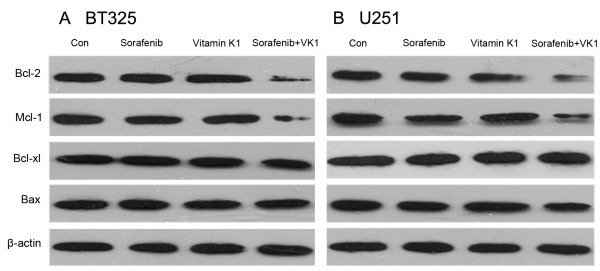
**Sorafenib in combination with vitamin K1 (VK1) modulates the expression of Bcl-2 protein family in BT325 (A) and U251 (B) cells.** The combination of the both agents exhibited strong synergistic action by inhibiting expression of Bcl-2 and Mcl-1 proteins, but showed little effects on expression of Bcl-xl and Bax proteins. Cells were treated with sorafenib (2.5 μM), VK1 (50 μM), or their combination for 24 h and β-actin was used as an internal control.

Because the intrinsic pathway of apoptosis is controlled by Bcl-2 family proteins, and cell death is regulated by the balance between proapoptotic (for example, Bax) and antiapoptotic (for example, Bcl-2, Bcl-xl, Mcl-1) pathways, we also detected the effect of combination drug treatment on such proteins. As shown in Figure 5, decreased levels of the antiapoptotic proteins Mcl-1 and Bcl-2 were observed after exposure to combination sorafenib (2.5 μM) plus VK1 (50 μM) in BT325 and U251 cells, but not observed after exposure to individual agents alone. There was little change in Bcl-xl or Bax levels.

## Discussion

The combination of nutritional factors with anticancer agents might be a promising strategy for improving the efficacy of chemotherapy [19,20]. In the present study, we reported that VK1 had the potential to enhance the antitumor activity of sorafenib in the glioma cell lines BT325 and U251. The effects of the agents on the inhibition of proliferation of glioma cells were measured by MTT assay. The results indicated that VK1 is safe for use in cells up to 100 μM, and sorafenib exerted a dose-dependent effect on cell growth inhibition. In addition, we found that low dose of sorafenib (2.5 μM) plus VK1 (0 to 100 μM) exerted synergistic efficacy in inhibiting cell growth in a time-dependent and dose-dependent manner. To observe the effect of the combined agents on cell apoptosis, we also performed apoptosis analysis using flow cytometry assays and DAPI staining. Our results showed that a combination of sorafenib (2.5 μM) with VK1 (50 μM) induced apoptosis and caused typical apoptotic morphology changes, including condensed chromatin as well as nuclear fragmentation.

The mechanisms by which combination sorafenib and VK1 synergistically induce apoptosis in glioma cells appear to be complex. The upregulation of the Raf/MEK/ERK cascade is one of the principal Ras-regulated pathways, and has been proven to be associated with glioma cell proliferation, survival and migration [6]. It has been suggested that downregulation of the Raf/MEK/ERK pathway may be of great promise as a target for preventing tumor cell growth [8,9]. In the present study, western blotting analysis showed that low concentrations of sorafenib (2.5 μM) or VK1 (50 μM) when used as single agents did not inhibit phospho-MEK and phospho-ERK levels, whereas the combination of both agents had a strong synergistic action to inhibit phosphorylation of MEK and ERK. These results suggest that the synergistic induction of cell apoptosis by combination of sorafenib and VK1 is, at least partly, attributed to inhibition of the Raf/MEK/ERK signaling pathway.

One important target for chemotherapy is programmed cell death, and this is determined by the balance of proapoptotic and antiapoptotic proteins. We also detected the Bcl-2 protein family, which regulates the mitochondrial pathway of apoptosis [21,22]. The Bcl-2 family is composed of antiapoptotic (for example, Bcl-2, Mcl-1 and Bcl-xl) and proapoptotic (for example, Bax) proteins. The antiapoptotic Bcl-2 protein targets intracellular organelles such as the endoplasmic reticulum, outer mitochondrial and nuclear membranes, and then modulates tumor cells responses to apoptosis [23]. Mcl-1 is another highly expressed antiapoptotic protein in malignant tumors and has been implicated in resistance to chemotherapy through a number of signaling pathways [24]. It has also been reported that ERK-mediated phosphorylation is an important regulator of Mcl-1 stability, and downregulation of Mcl-1 is one of the main antitumor effect of sorafenib, either alone or in combination with other agents, on several kinds of tumor cells [25,26]. In our study, the combination of low concentration sorafenib (2.5 μM) and VK1 (50 μM) exhibited strong synergistic action by inhibiting protein expression of Bcl-2 and Mcl-1, leading to induction of cell apoptosis. However, there were no obvious changes in the expression of Bcl-xl and Bax proteins. These results suggested that the combination of sorafenib with VK1 induced apoptosis in BT325 and U251 cells through downregulating proapoptotic proteins Bcl-2 and Mcl-1.

## Conclusions

The present study indicated that VK1 could enhance sorafenib-induced growth inhibition and apoptosis of glioma cells, and this synergistic effectiveness was associated with the downregulation of the Raf/MEK/ERK signaling pathway. These results make the combination of sorafenib and VK1 a promising therapeutic strategy against human malignant gliomas.

## Competing interests

The authors declare that they have no competing interests.

## Authors’ contributions

WD and Q-jZ designed the study and wrote the article. WD and J-rZ conducted the experiments and carried out the statistical analyses. D-lW and KG assisted with experiments and manuscript preparation. All authors read and approved the final manuscript.
